# Different effects of meteorological factors on hand, foot and mouth disease in various climates: a spatial panel data model analysis

**DOI:** 10.1186/s12879-016-1560-9

**Published:** 2016-05-26

**Authors:** Chao Wang, Kai Cao, Yingjie Zhang, Liqun Fang, Xia Li, Qin Xu, Fangfang Huang, Lixin Tao, Jin Guo, Qi Gao, Xiuhua Guo

**Affiliations:** Department of Epidemiology and Health Statistics, School of Public Health, Capital Medical University, No.10 Xitoutiao, Youanmen Wai Street, Fengtai District Beijing, 100069 PR China; Beijing Municipal Key Laboratory of Clinical Epidemiology, No.10 Xitoutiao, Youanmen Wai Street, Fengtai District Beijing, 100069 PR China; Department of Statistics and Information, Beijing Centers for Disease Control and Prevention, No.16, Hepingli Middle Street, Dongcheng District Beijing, 100013 PR China; National Center for Public Health, Surveillance and Information Services, Chinese Center for Disease Control and Prevention, No. 155 Changbai Street, Changping District Beijing, 102206 PR China; State Key Laboratory of Pathogen and Biosecurity, Beijing Institute of Microbiology and Epidemiology, No. 20 Dongda Street, Fengtai District Beijing, 100071 PR China; The Graduate Entry Medical School, University of Limerick, Limerick, Ireland

**Keywords:** Hand, foot and mouth disease, Spatial panel data model, Meteorological factors, Climate type

## Abstract

**Background:**

Major outbreaks of hand, foot and mouth disease (HFMD) have been reported in China since 2008, posing a great threat to the health of children. Although many studies have examined the effect of meteorological variables on the incidence of HFMD, the results have been inconsistent. This study aimed to quantify the relationship between meteorological factors and HFMD occurrence in different climates of mainland China using spatial panel data models.

**Methods:**

All statistical analyses were carried out according to different climate types. We firstly conducted a descriptive analysis to summarize the epidemic characteristics of HFMD from May 2008 to November 2012 and then detected the spatial autocorrelation of HFMD using a global autocorrelation statistic (Moran’s I) in each month. Finally, the association between HFMD incidence and meteorological factors was explored by spatial panel data models.

**Results:**

The 353 regions were divided into 4 groups according to climate (G1: subtropical monsoon climate; G2: temperate monsoon climate; G3: temperate continental climate; G4: plateau mountain climate). The Moran’s I values were significant with high correlations in most months of group G1 and G2 and some months of group G3 and G4. This suggested the existence of a high spatial autocorrelation with HFMD. Spatial panel data models were more appropriate to describe the data than fixed effect models. The results showed that HFMD incidences were significantly associated with average atmospheric pressure (AAP), average temperature (AT), average vapor pressure (AVP), average relative humidity (ARH), monthly precipitation (MP), average wind speed (AWS), monthly total sunshine hours (MSH), mean temperature difference (MTD), rain day (RD) and average temperature distance (ATD), but the effect of meteorological factors might differ in various climate types.

**Conclusions:**

Spatial panel data models are useful and effective when longitudinal data are available and spatial autocorrelation exists. Our findings showed that meteorological factors were related to the occurrence of HFMD, which were also affected by climate type.

## Background

Hand, foot and mouth disease (HFMD) is a common infectious disease, which is mainly caused by the enteroviruses *coxsackie A16* and *enterovirus 71* [1]. In most cases, the disease is mild and self-limiting, but sometimes serious neurological and cardiopulmonary complications may occur, particularly among those aged 5 years and younger [2]. HFMD was listed as a notifiable Class-C communicable disease since May 2008 [3]. In recent years, the trend of HFMD outbreaks has increased among children in China, which is regarded as an important public health problem [4, 5].

Many studies have been performed to analyze the association between meteorological factors and HFMD, but the results have been inconsistent. It has been found that temperature and relative humidity were positively associated with HFMD in most studies [6–11], whereas studies in Japan found that the number of days per week with an average temperature above 25°C was negatively associated with HFMD incidence [12]. Wind velocity was found to be positively associated with HFMD in Ma [13] and Li’s studies [13], but in Huang’s publication [8], no relationship was found between wind speed and HFMD. Furthermore, Wang [10] found a negative association. Other possible risk factors (total sunshine, difference in temperature, atmospheric pressure, vapor pressure, etc.) were examined in a few studies and require further research.

Most previous studies have not considered temporal or spatial effects (Multiple Linear Regression Model [13]/Negative Binomial Regression [14]/Generalized Additive Poisson Model [15]), or merely focused on the spatial dimension (Geographically Weighted Regression Models (GWR) [16]) or on time dimension (time-series analysis [8]) approaches, which might cause loss of information by ignoring the heterogeneity in both time and space and result in different conclusions.

Compared to traditional methods on the basis of time-series or cross-sectional data alone, spatial panel data models can control for both spatial dependency and unknown heterogeneity [17, 18]. In this study, spatial panel data models were used to explore the relationship between meteorological variables and HFMD incidence in 353 regions of mainland China according to climate types.

## Methods

### Data collection

#### Study area

**Fig. 1:**
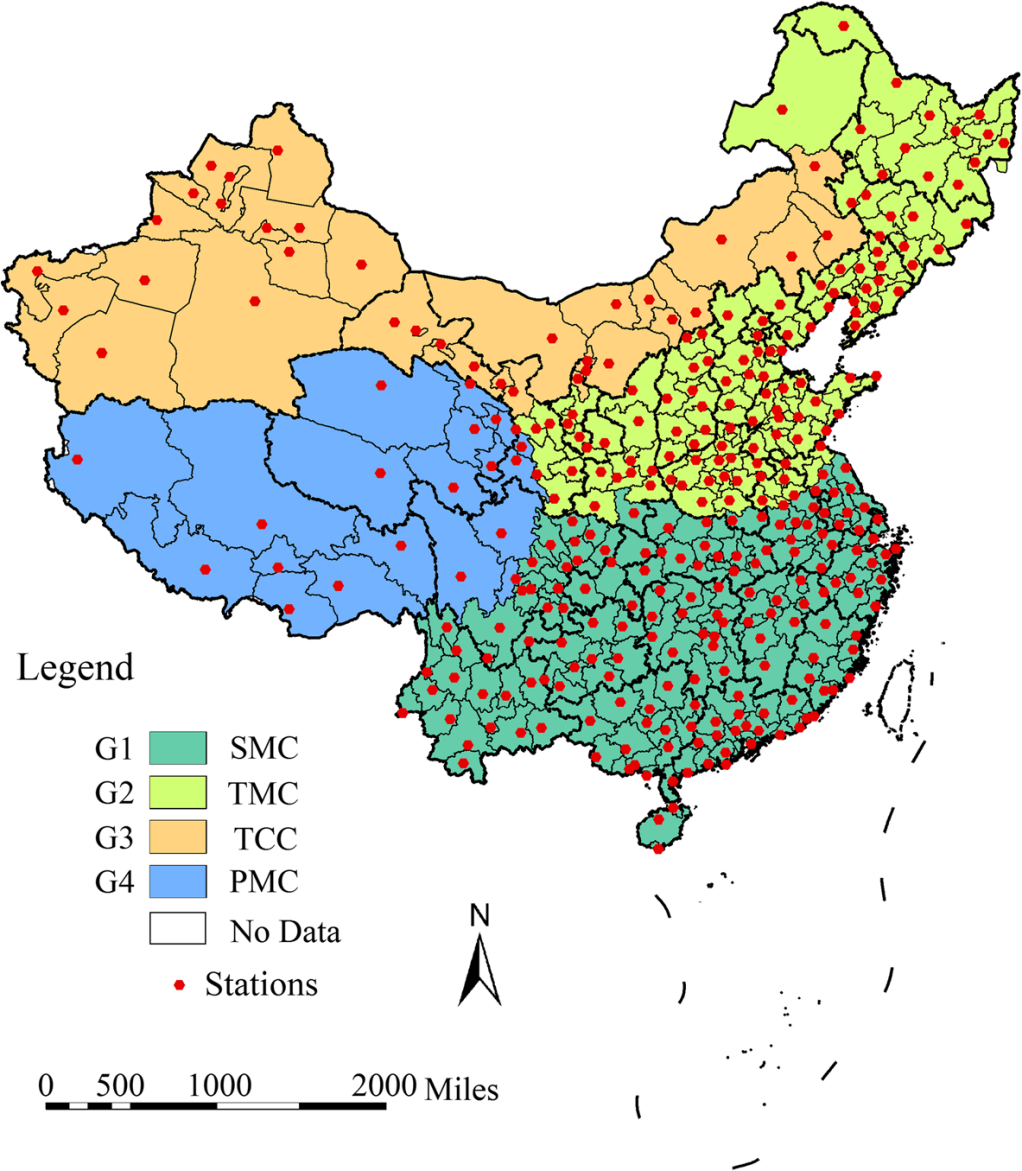
Climate types distribution and locations of meteorological monitoring stations in mainland China. SMC (Group 1, G1): subtropical monsoon climate; TMC (Group 2, G2): temperate monsoon climate; TCC (Group 3, G3): temperate continental climate; PMC (Group 4, G4): and plateau mountain climate

Thirty-one provinces of mainland China were divided into 353 regions in this study. There were four main types of climate in mainland China. All the data were grouped by climate types, as shown in Fig. 1: subtropical monsoon climate (SMC, G1), temperate monsoon climate (TMC, G2), temperate continental climate (TCC, G3) and plateau mountain climate (PMC, G4).

### Surveillance data of HFMD

Data of monthly reported HFMD cases in each region in mainland China from May 2008 to November 2012 were obtained from the National Center for Public Health Surveillance and Information Services, at the China Center for Disease Control and Prevention (China CDC). All cases were diagnosed according to the clinical criteria provided in a guidebook published by the National Health and Family Planning Commission of the People’s Republic of China in 2008 [19]: with or without fever, a probable case of HFMD was defined as a patient with a papular or vesicular rash on or in the hands, feet, mouth, and/or buttocks. A confirmed case was defined as a probable case with laboratory evidence of enterovirus infection. The demographic data for each region were obtained from the National Bureau of Statistics of China [20]. The ethical approval and the consent from each individual subject was not required because we used only aggregated data (number of cases for each county in months) but not any individualized data in this study.

### Meteorological data

Meteorological data from 328 monitoring stations that were nearest to the centers of 353 regions were obtained from the China Meteorological Data Sharing Service System (http://cdc.nmic.cn/home.do), which was publicly accessible. Monthly meteorological variables in this study included average atmospheric pressure (AAP), average temperature (AT), average vapor pressure (AVP), average relative humidity (ARH), monthly precipitation (MP), average wind speed (AWS), monthly total sunshine hours (MSH), mean temperature difference (MTD), monthly rainfall days (MRD), and average temperature distance (ATD).

### Statistical analysis

#### Global spatial autocorrelation analysis

The autocorrelation statistic (global Moran’s I) [21] was used to detect the global spatial autocorrelation of reported HFMD incidences in the study area by climate types. The significance of Moran’s I was assessed by employing Monte Carlo randomization. A higher positive Moran’s I value with a statistically significant *p*-value (*P* < 0.05) indicates that the values of neighboring areas tend to cluster [22]. ArcGIS 10.1 (*ESRI Inc. Redlands, CA, USA*) was used to perform the analysis.

### Spatial panel data models

Spatial panel data typically refer to data containing continuous observations of a number of spatial units. Spatial panel models, which could address data with spatial dependence and also enable researchers to consider spatial and/or temporal heterogeneity, were used to examine the role of different meteorological factors in this study. Spatial panel data models are more informative and contain more variation and less collinearity between the variables compared with cross-sectional or time series models [17].

The basic form of the simple panel data model with a spatial and temporal specific effect is:

$$ {y}_{it}={\mu}_i+{\gamma}_t+{X}_{it}\beta +{\varepsilon}_{it} $$

where *i* and *t* are indices for the cross-sectional dimension (spatial units) and time dimension (time periods), respectively; *y*_*it*_ is the dependent variable at *i* and *t*; *X*_*it*_ is the group of explanatory variables; β is the vector of regression coefficients that explains the relationship between *X*_*it*_ and *y*_*it*_; *ε*_*it*_ is an independently and identically distributed error term with zero mean and variance σ^2^; *μ*_*i*_ denotes a spatial specific effect and *γ*_*t*_ represents temporal specific effects. The spatial and/or temporal specific effects may be treated as fixed effects or random effects. A random effect is appropriate if a certain number of individuals is randomly sampled from a large population. The fixed effect model is favored [23–25] when the regression analysis is applied to a precise set of regions. For this reason, because our data contained all regions of the study area, we established fixed effects panel data models that included spatial error autocorrelation or a spatially lagged dependent variable. We also compared the random effects specification against fixed effects specification by Housman’s specification test [26, 27], which suggested that fixed effect specification was more appropriate.

The simple panel data models with specific effects can be extended to the spatial lag (including spatially lagged dependent variables) and the spatial error model (including spatial error autocorrelation terms). The dependent variable in the spatial lag model depends on the dependent variable observed in neighboring units [17]. The spatial lag model with spatial and temporal fixed effects could be specified as follows [28]:

$$ {y}_{it}={\mu}_i+{\gamma}_t+\rho {\displaystyle \sum_{j=i}^N{W}_{ij}{y}_{ij}+{X}_{it}\beta +{\varepsilon}_{it}} $$

The spatial error model assumed that the effect of spatial correlation is a representation of the ignored variable. The model with spatial and temporal fixed effects could be specified as below [28]:

$$ {y}_{it}={\mu}_i+{\gamma}_t+{X}_{it}\beta +{\phi}_{it} $$

$$ {\phi}_{it}=\lambda {\displaystyle \sum_{j=i}^N{W}_{ij}{\phi}_{ij}+{\varepsilon}_{it}} $$

where W is an N × N positive non-stochastic spatial weight matrix; ρ is the spatial autoregressive coefficient and λ is usually called the spatial autocorrelation coefficient.

Likelihood ratio (LR) tests were used to determine the spatial and/or time-period fixed effects in the extension of the model. The Lagrange multiplier (LM) and robust Lagrange multiplier (robust LM) test were used to determine whether the spatial lag model and/or the spatial error model are more appropriate than a simple panel data model to describe the data. The spatial error model is more appropriate if the LM error is more significant than the LM lag and the robust LM error test is significant and the robust LM lag is not significant and vice versa. R^2^ and log-likelihood were the commonly used effective criteria to evaluate the model [29–31]. Matlab R2014a (*Mathworks Inc., Natick, MA, USA*) was used to perform the analysis of the spatial panel data models, LR, and LM tests.

## Results

### Basic description

**Fig. 2:**
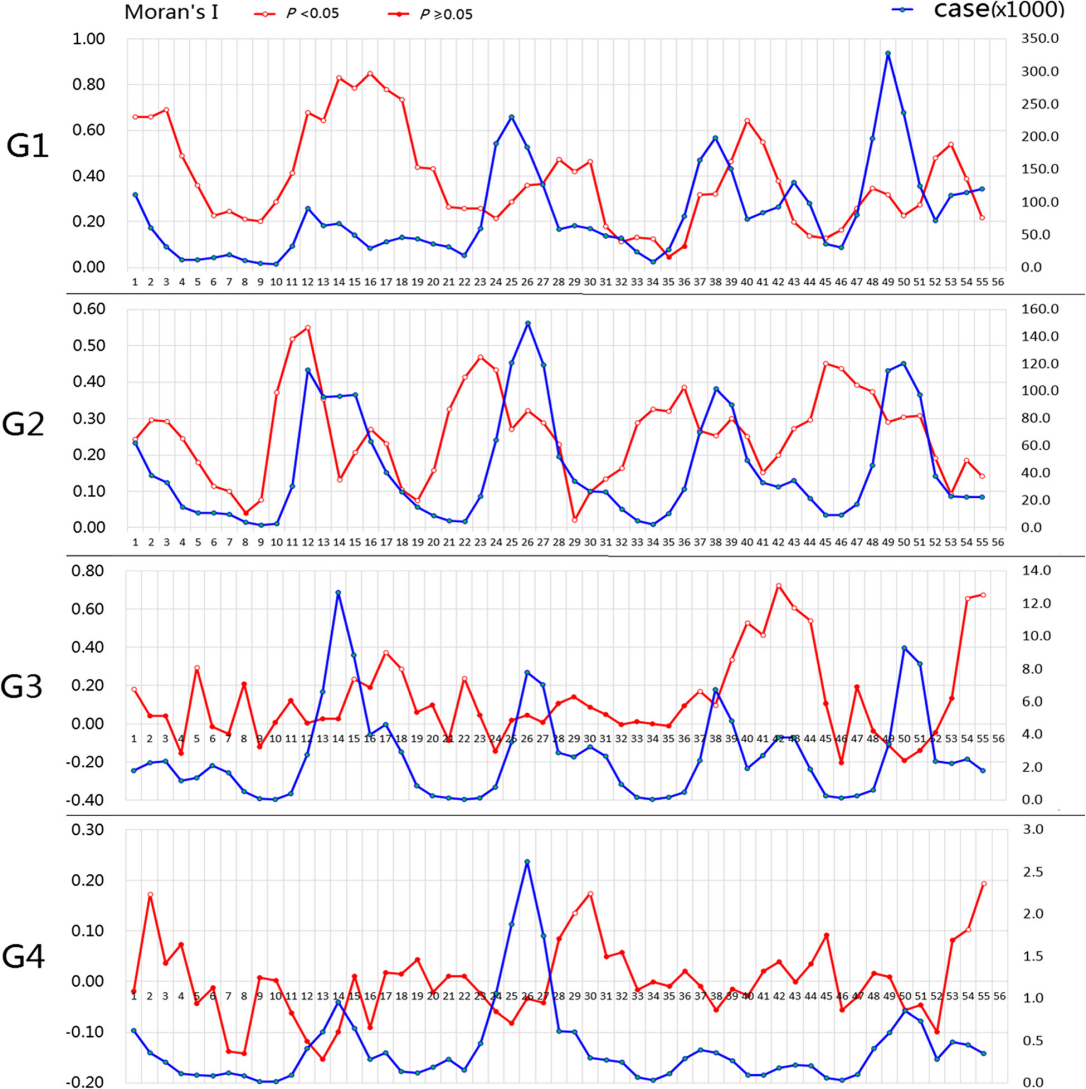
The number of reported cases and Moran’I indices (the spatial autocorrelation tests) for each month in different groups

A total of 7,061,525 HFMD cases were reported in 353 regions of mainland China from May 2008 to November 2012. The number of cases of HFMD per month ranged from 0 to 11,730 in each region. The number of reported cases for each month is shown in Fig. 2, which indicates a potential seasonality of the incidence of HFMD, since more cases occurred in April-July.

### Spatial autocorrelation of HFMD incidence

The spatial autocorrelation test for each month is shown in Fig. 2. The figure demonstrates that a high global spatial autocorrelation of HFMD was detected at the regional level in mainland China within each epidemic month during May 2008 to November 2012 in group G1 (Moran’s I from 0.046 to 0.848) and group G2 (Moran’s I from 0.020 to 0.550). In group G3 and group G4, the global Moran’s I value was only significant in some months. Because of the existence of a high spatial dependency on the occurrence of HFMD, a spatial autocorrelation should be included in the regression model and thus, the panel data models were more appropriate.

### Spatial panel data models

Logarithmic transformation of the reported incidence was used due to the distribution of the reported disease incidence having a non-normal distribution. Because of some zeroes in the dependent variable, we assumed that there were 0.5 cases in the zones with no reported cases before calculating the incidence and the logarithmic transformation. Table 1 shows the basic descriptive statistics for meteorological variables and dependent variables (logarithmic transformation of the reported incidence) in each group.

**Table 1:** Descriptive statistics for meteorological variables and dependent variables (Y)

variables	G1 (172 regions)		G2 (127 regions)		G3 (34 regions)		G4 (20 regions)	
	Mean(SD)	Median (interquartile range)	Mean(SD)	Median (interquartile range)	Mean(SD)	Median (interquartile range)	Mean(SD)	Median (interquartile range)
Y	−4.36(0.71)	−4.28(−4.77,-3.85)	−4.65(0.83)	−4.52(−5.17,-4.03)	−5.02(1.00)	−4.88(−5.74,-4.23)	−5.34(0.71)	−5.52(−5.89,-4.89)
AAP	974.27(63.13)	999.80(969.60,1009.70)	972.14(57.44)	996.50(956.05,1009.50)	895.15(53.07)	889.65(858.90,933.03)	695.40(87.98)	685.25(625.55,729.80)
AT	18.83(7.74)	20.15(12.70,25.40)	11.28(11.83)	14.00(2.35,21.3)	8.99(12.90)	11.30(−2.30,20.30)	5.83(8.74)	7.00(−0.68,12.80)
AVP	17.62(7.95)	17.00(10.50,24.60)	10.92(7.79)	9.10(4.10,17.00)	6.44(4.33)	5.50(2.60,9.90)	5.85(4.50)	4.80(2.10,8.48)
ARH	74.22(8.35)	75.00(70.00,80.00)	63.18(12.93)	64.00(54.00,73.00)	48.93(14.04)	48.00(38.00,59.00)	53.05(17.99)	56.00(39.00,67.00)
MP	115.57(116.65)	83.60(33.20,161.68)	55.37(68.67)	30.70(9.00,77.30)	17.38(26.82)	6.95(1.10,22.13)	46.20(64.02)	23.95(2.5,71.08)
AWS	1.94(0.86)	1.80(1.30,2.30)	2.24(0.91)	2.10(1.70,2.60)	2.24(0.78)	2.10(1.70,2.70)	1.87(0.82)	1.70(1.30,2.20)
MSH	139.12(60.42)	136.90(95.10,182.80)	189.24(51.18)	190.20(155.20,224.70)	246.24(59.85)	250.80(209.38,290.75)	212.39(55.90)	217.55(183.95,248.83)
MTD	7.92(2.14)	7.70(6.60,9.00)	10.38(2.39)	10.30(8.60,11.90)	12.88(2.42)	12.90(11.40,14.40)	13.43(3.07)	13.50(11.60,15.48)
RD	11.57(5.38)	11.00(8.00,15.75)	7.33(4.48)	7.00(4.00,10.00)	4.95(4.08)	4.00(2.00,7.00)	10.47(7.97)	9.00(3.00,17.00)
ATD	0.47(1.36)	0.50(−0.30,1.2)	0.37(1.49)	0.40(−0.40,1.30)	0.70(1.78)	0.90(−0.20,1.90)	0.96(1.20)	0.95(0.30,1.70)

Table 2 shows the results to determine which specific fixed effect and which type of spatial dependency term should be included in the model for each group. The LR tests showed that the extension of the model with both spatial and time-period fixed effects was more suitable (*P* < 0.001). LM and robust LM test results demonstrated that the spatial lag models were more appropriate than the spatial error models. Overall, the test results implied that the spatial lagged model with spatial and time-period fixed effects was more appropriate to process the data.

**Table 2:** Results of tests to determine specific fixed effects and spatial dependency terms for each group

Type of test		G1	G2	G3	G4
LR_tests of fixed effects	Spatial fixed effects	6732.35**	2607.12**	2021.77**	415.62**
	Temporal fixed effects	7461.23**	2729.13**	753.29**	309.58**
LM_Tests	LM Lag test	1506.82**	2939.76**	47.13**	6.42*
	Robust LM Lag test	6.84**	186.74**	20.45**	7.11**
	LM Error test	1500.82**	2773.34**	39.29**	4.62*
	Robust LM Error test	0.84	20.32**	12.61**	5.30*

**: *P* < 0.01; *: *P* < 0.05

The results of the three models (fixed effects model, spatial lag panel model and spatial error panel model) for each group are shown in Table 3. It can be seen in Table 3 that the spatial lag panel model and the spatial error panel model were better than the classic fixed effects model, and the spatial lag model is more appropriate than the spatial error model at comparing the values of R^2^ and the log-likelihood in each group. Both the spatial autoregressive coefficient (*ρ* = 0.452, 0.615, 0.241 and 0.201 for G1, G2, G3, and G4, respectively) and the spatial autocorrelation coefficient (*λ* = 0.450, 0.616, 0.178 and 0.092 for G1, G2, G3, and G4, respectively) were positive and statistically significant, suggesting the HFMD incidence of a spatial unit correlates positively with the incidence of surrounding spatial units and unmeasured variables.

**Table 3:** Results of the fixed effects model (FEM), spatial lag (SLM) and spatial error (SEM) fixed effects panel models in each group

Variable	G1			G2			G3			G4		
	FEM	SEM	SLM	FEM	SEM	SLM	FEM	SEM	SLM	FEM	SEM	SLM
AAP	0.568**	0.935**	0.704**	0.816**	0.167	0.288	0.521	0.405	0.382	1.653	1.866	1.494
AT	−0.096*	−0.044	−0.020	0.619**	0.498**	0.236**	0.682**	0.520**	0.529**	0.461**	0.425**	0.379*
AVP	0.208**	0.233**	0.157**	−0.497**	−0.362**	−0.227**	0.098	0.134*	0.099	0.584**	0.548**	0.512**
ARH	−0.007	−0.031	−0.013	0.287**	0.094**	0.110**	−0.030	−0.050	−0.043	−0.142	−0.135	−0.132
MP	0.011	0.010	0.008	0.013	−0.002	0.004	−0.012	−0.002	−0.007	−0.130**	−0.122**	−0.119**
AWS	−0.033*	−0.021	−0.028*	−0.033	−0.005	−0.013	−0.006	−0.001	0.002	−0.263**	−0.262**	−0.253**
MSH	0.046**	0.032*	0.034**	0.014	0.006	0.002	−0.100**	−0.107**	−0.097**	−0.144*	−0.142*	−0.135*
MTD	−0.074**	−0.056**	−0.048**	0.061**	0.027	0.033	0.030	0.029	0.026	0.056	0.070	0.062
RD	−0.003	0.006	0.002	0.007	0.023	0.007**	0.044	0.036	0.037	−0.019	−0.012	−0.014
ATD	0.040**	0.014	0.016	0.073**	−0.009	0.026**	−0.022	−0.007	−0.014	0.001	0.015	0.008
Lambda(λ)	-	0.450**	-	-	0.616**	-	-	0.178**	-	-	0.092*	-
Rho(ρ)	-	-	0.452**	-	-	0.615**	-	-	0.241**	-	-	0.201**
R^2^	0.765	0.764	0.803	0.700	0.692	0.802	0.789	0.789	0.797	0.565	0.565	0.570
Log-likelihood	−3283.500	−2676.000	−2675.180	−4390.400	−3299.800	−3280.479	−1200.400	−1181.126	−1178.103	−729.547	−727.100	−726.281

**: *p* < 0.01; *: *p* < 0.05

Different significant factors were found in different groups. The average atmospheric pressure (AAP), average vapor pressure (AVP), monthly total sunshine hours (MSH) and mean temperature difference (MTD) had a significant correlation with the HFMD incidence in the three models for regions, and average wind speed (AWS) was also significant in the spatial lag model for south/east China. Average temperature (AT), average vapor pressure (AVP), average relative humidity (ARH), rain day (RD) and average temperature distance (ATD) were risk factors in the spatial lag model in regions with a temperate monsoon climate (TMC). Average temperature (AT) has a significant positive correlation with the HFMD incidence, but monthly total sunshine hours (MSH) was found to have a significant negative correlation in both G3 and G4. Monthly precipitation (MP) and average wind speed (AWS) also had a significant negative association with the HFMD incidence in G4, although average vapor pressure (AVP) was positively significant. The collected variables and the spatial neighborhood effects could jointly explain approximately 80.0 % of the variation of the HFMD incidence in the first 3 groups (G1 [R^2^ = 0.803], G2 [R^2^ = 0.802] and G3 [R^2^ = 0.797]) and 57.0 % in group G4 (R^2^ = 0.570). We can also find that most coefficients of spatial panel models were smaller than the fixed effect model, which indicated that if the spatial autocorrelation is ignored, the effects of meteorological factors would be overestimated.

## Discussion

The relationship between HFMD and meteorological factors was quantified using spatial panel data models based on longitudinal data from 353 regions of mainland China according to climate type from May 2008 to November 2012.

According to Elhorst [17], spatial panel data models contain more variation and less co-linearity among variables and are more informative than purely cross-sectional models or time-series models. The model tests justified the spatial and temporal fixed effects, which indicate that spatial and temporal heterogeneity do affect the robustness of statistical models, and taking the panel structure and spatial autocorrelation terms in the model simultaneously result in a better fit for the data [10]. Previous studies have confirmed the existence of spatial correlations in the incidence of HFMD in various regions, which is consistent with our study [32–35]. Both the spatial autoregressive coefficient in the spatial lag panel model and the spatial autocorrelation coefficient in the spatial error panel model are positive and statistically significant. This further indicates the importance and necessity to include the spatial correlation in exploring the risk factors of HFMD.

These results show that every meteorological factor might have an association with HFMD incidence, and the effect may differ with different climates. A positive relationship between temperature and HFMD incidence (in group G2, G3 and G4) as well as between relative humidity (in group G2) and HFMD incidence was found in our study, which was consistent with the finding of other recent studies [8–10, 13–16]. Average wind speed has a negative association with HFMD incidence (in group G1 and G4) in this study, which is inconsistent with Ma’s, Li’s and Yong Huang’s studies [8, 13, 14]. A negative effect was observed for sunshine at lag days 3–4 in Wu’s study [15], which is consistent with the results in groups G3 and G4 in our research. Deng’s study [9] showed a positive correlation between sunshine and HFMD, which was also consistent with our result in group G1. The differences in the results may be caused by different climate types, which need further research. AAP was found to be positively significant with HFMD in group G1 in this study, which was different from Li’s study in Guangzhou [14]. In Wang’s research [10], vapor pressure had a negative correlation with HFMD, which is consistent with our findings in G2. We also found opposite results in G1 and G4. We also found that monthly precipitation (MP) and mean temperature difference (MTD) were negatively correlated with HFMD in G4 and G1, respectively. Both rain day (RD) and average temperature distance (ATD) had a positive relationship with HFMD in G2, but not in G1, G3 and G4.

There are some limitations in this study. Although the spatial fixed effect and temporal fixed effect were included in the spatial panel models, the models did not consider the effect of space-time interaction, and we assumed that the effect of meteorological variables on HFMD was consistent in all regions. However, the population and climate distribution vary greatly in the world’s third biggest country. It would be difficult to keep the spatial stationarity assumption in such a complicated area. The geographical weighted regression model (GWR) or random coefficients models would be more useful to explore the local effect of factors of interest in the future. We selected the month as the temporal scale; more precise results or lag effects of meteorological factors may have been attained if detailed data were obtained and used for the analysis. Another limitation is that other factors, such as social/economic status, which may affect the HFMD, were not quantified in this study.

## Conclusions

Spatial panel data models are useful and effective when longitudinal data are available and spatial autocorrelation exists. This study provides quantitative evidence that the incidence of HFMD could be affected by average atmospheric pressure, average temperature, average vapor pressure, average relative humidity, monthly precipitation, average wind speed, monthly total sunshine hours, mean temperature difference, rain day and average temperature distance. The effects of meteorological factors may be different in different climate types. This can facilitate a better understanding of epidemic trends and a preparedness for HFMD prevention and control strategies.

## Abbreviations

AAP: average atmospheric pressure
ARH: average relative humidity
AT: average temperature
ATD: average temperature distance
AVP: average vapor pressure
AWS: average wind speed
China CDC: China Center for Disease Control and Prevention
GWR: geographically weighted regression models
HFMD: hand, foot and mouth disease
LM: lagrange multiplier
LR: likelihood ratio
MP: monthly precipitation
MSH: monthly total sunshine hours
MTD: mean temperature difference
PMC: plateau mountain climate
RD: rain day
Robust LM: robust Lagrange multiplier
SMC: subtropical monsoon climate
TCC: temperate continental climate
TMC: temperate monsoon climate
